# Comparative genomic hybridization detects many recurrent imbalances in central nervous system primitive neuroectodermal tumours in children

**DOI:** 10.1038/sj.bjc.6690293

**Published:** 1999-04

**Authors:** H Avet-Loiseau, A-M Vénuat, M-J Terrier-Lacombe, A Lellouch-Tubiana, M Zerah, G Vassal

**Affiliations:** Laboratory of Hematology, Institut de Biologie, 9 quai Moncousu, Nantes, Cédex 1, 44093, France; Laboratory of Genetics and Cytogenetics, Department of Pathology, Department of Pediatric Oncology and CNRS URA 147, Institut Gustave Roussy, 39 rue Camille Desmoulins, Villejuif, Cédex, 94805, France; Department of Pathology, Department of Pediatric Neurosurgery, Hopital Necker-Enfants Malades, 149 rue de Sevres, Paris, Cedex 15, 75743, France

**Keywords:** CGH, PNET, medulloblastoma, FISH, cytogenetics

## Abstract

A series of 23 children with primitive neuroectodermal tumours (PNET) were analysed with comparative genomic hybridization (CGH). Multiple chromosomal imbalances have been detected in 20 patients. The most frequently involved chromosome was chromosome 17, with a gain of 17q (11 cases) and loss of 17p (eight cases). Further recurrent copy number changes were detected. Extra copies of chromosome 7 were present in nine patients and gains of 1q were detected in six patients. A moderate genomic amplification was detected in one patient, involving two sites on 3p and the whole 12p. Losses were more frequent, and especially involved the chromosomes 11 (nine cases), 10q (eight cases), 8 (six cases), X (six patients) and 3 (five cases), and part of chromosome 9 (five cases). These recurrent chromosomal changes may highlight locations of novel genes with an important role in the development and/or progression of PNET. © 1999 Cancer Research Campaign


*British Journal of Cancer* (1999) **79**(11/12), 1843–1847
© 1999 Cancer Research Campaign
Article no. bjoc.1998.0293

### Comparative genomic hybridization detects many recurrent imbalances in central nervous system primitive neuroectodermal tumours in children

**H Avet-Loiseau[1], A-M Vénuat[2], M-J Terrier-Lacombe[3], A Lellouch-Tubiana[5], M Zerah[6] and G Vassal[4]**

[1]Laboratory of Hematology, Institut de Biologie, 9 quai Moncousu, 44093 Nantes Cédex 1, France; [2]Laboratory of Genetics and Cytogenetics, [3]Department of Pathology, [4]Department of Pediatric Oncology and CNRS URA 147, Institut Gustave Roussy, 39 rue Camille Desmoulins, 94805 Villejuif Cédex, France; [5]Department of Pathology, [6]Department of Pediatric Neurosurgery, Hopital Necker-Enfants Malades, 149 rue de Sevres, 75743 Paris Cedex 15, France

**Summary** A series of 23 children with primitive neuroectodermal tumours (PNET) were analysed with comparative genomic hybridization (CGH). Multiple chromosomal imbalances have been detected in 20 patients. The most frequently involved chromosome was chromosome 17, with a gain of 17q (11 cases) and loss of 17p (eight cases). Further recurrent copy number changes were detected. Extra copies of chromosome 7 were present in nine patients and gains of 1q were detected in six patients. A moderate genomic amplification was detected in one patient, involving two sites on 3p and the whole 12p. Losses were more frequent, and especially involved the chromosomes 11 (nine cases), 10q (eight cases), 8 (six cases), X (six patients) and 3 (five cases), and part of chromosome 9 (five cases). These recurrent chromosomal changes may highlight locations of novel genes with an important role in the development and/or progression of PNET.

**Keywords:** CGH; PNET; medulloblastoma; FISH; cytogenetics

Primitive neuroectodermal tumours (PNET) are the most frequent primary malignant childhood brain tumours (Farwell et al, 1977; Bigner et al, 1988). Medulloblastomas represent the majority of cerebral PNET and are located in the cerebellum. Because of difficulties in generating adequate metaphases, cytogenetic data in these tumour types are still limited. The most frequent abnormality is isochromosome i(17q), found in approximately 30% of cases analysed with conventional cytogenetics (Bigner et al, 1988; Griffin et al, 1988; Biegel et al, 1989; Karnes et al, 1992; Vagner-Capodano et al, 1992; Neumann et al, 1993; Fujii et al, 1994). This high incidence of i(17q) was confirmed in a molecular cytogenetic study, which also showed deletions of 17p in 44% of patients (Biegel et al, 1995). Other studies using molecular approaches confirmed this frequent loss of 17p, and localized a hot-spot of loss of heterozygosity at 17p13.3, a locus telomeric to the p53 gene (Biegel et al, 1992; Cogen et al, 1992; McDonald et al, 1994; Batra et al, 1995). A very recent deletion mapping study localized a common chromosomal disruption within a more centromeric region, at 17p11.2 (Scheurlen et al, 1997). Other recurrent abnormalities have been described, including structural aberrations of chromosomes 1, 3, 6, 11, 16 and X, loss of chromosome 22 and gains of chromosomes 6 and 8 (Farwell et al, 1977; Bigner et al, 1988, 1990; Griffin et al, 1988; Callen et al, 1989; Karnes et al, 1992; Neumann et al, 1993; Fujii et al, 1994). Few gene amplifications have been reported, involving *MYC*, *MYCN* or *EGFR* (Rouah et al, 1989; Wasson et al, 1990; Fuller and Bigner, 1992; Badiali et al, 1995), and more recently the 5p15 and 11q22 chromosomal regions (Reardon et al, 1997).

The recently developed technique of comparative genomic hybridization (CGH) allows a genome-wide screening for unbalanced abnormalities, without the requirement for tumour metaphases (reviewed in Kallioniemi et al, 1994). Only two studies using this approach in PNET have previously been reported (Schutz et al, 1996; Reardon et al, 1997). In order to extend our knowledge on recurrent imbalances in this disease, we analysed frozen tumour samples obtained from 23 children with either medulloblastoma (21 patients) or supratentorial PNET (two patients). We found many recurrent abnormalities, especially gains of 17q, 1q and 7, and losses of chromosomes 3, 8, 10q, 11, 21 and X.

### MATERIALS AND METHODS

### Patients

Primary brain tumour tissue samples were obtained from 23 patients with a PNET. Samples were obtained at diagnosis in 22 patients and at time of relapse in 1 patient, a 13-year-old boy with a non-metastatic medulloblastoma (patient 221) (Table 1). There were 11 girls and 12 boys with a median age of 7.5 years (range 9 months–13 years). Twenty-one patients had a cerebellar tumour, i.e. medulloblastoma, and two a supratentorial PNET. The disease was metastatic at diagnosis in nine patients with newly diagnosed medulloblastoma, as assessed by magnetic resurance imaging (MRI) and cerebrospinal fluid (CSF) analysis. Tissue samples were immediately frozen in liquid nitrogen upon tumour removal and stored at –80°C until analysis. For histological analysis, tissue

*Received 19 May 1998*
*Revised 25 August 1998*
*Accepted 18 September 1998*

*Correspondence to:* H Avet-Loiseau, Laboratory of Hematology, Biology Institute, 9 quai Moncousu, 44093 Nantes Cedex, France

**Table 1** Main clinical characteristics and CGH results of the 23 patients

| Patient no. | Sex | Age (years) | Histology | Tumour location | Metastasis | CGH results | |
|---|---|---|---|---|---|---|---|
| | | | | | | Gains | Losses |
| 89 | F | 3 | Medulloblastoma | Posterior fossa | No | 6, 8, 13, 17q | 4q, 10, 11, 21, X |
| 99 | F | 4 | PNET with glial component | Supratentorial | No | 1q, 2, 17 | |
| 107 | M | 9 | Medulloblastoma | Posterior fossa | No | 7, 17q | 2q14q33, 3, 11, 17p |
| 127 | M | 9.7 | Medulloblastoma | Posterior fossa | No | 1q, 2, 12q22qter, 17q | 3, 8, 10, 11, 17p |
| 161 | M | 4.1 | Medulloblastoma | Posterior fossa | No | 5, 7, 17q | 3, 8, 11, 15, 17p, 20 |
| 202 | M | 12.2 | Medulloblastoma | Posterior fossa | No | 7 | 2, 3, 8, 9pterq22, 13, 15, 20 |
| 208 | M | 4.2 | PNET with glial component | Supratentorial | No | | 9pterq22, 11q23qter |
| 221 | M | 13 | Medulloblastoma | Posterior fossa | * | 7, X | 8p, 10q, 11, 13 |
| 222 | M | 6.4 | Medulloblastoma | Posterior fossa | No | 3pterp24, 3p23p21, 12p | 17p |
| 223 | F | 12.7 | Medulloblastoma | Posterior fossa | No | | 6 |
| 228 | M | 8.9 | Medulloblastoma | Posterior fossa | No | 1q, 7, 17q | 9p21q21 |
| 234 | M | 12 | Medulloblastoma | Posterior fossa | No | 1p31qter, 4, 5, 7, 18 | 8, 9, 10, 11, 12p12q14, 16, 21 |
| 245 | F | 7.5 | Medulloblastoma | Posterior fossa | No | 1q31qter, 3q, 13, Xq | 3p, 10q, 14q21qter |
| 249 | F | 7.7 | Medulloblastoma | Posterior fossa | No | 4, 7, 17 | 8, 10, 11, 15q11q15, X |
| 77 | F | 6.6 | Medulloblastoma | Posterior fossa | Yes | 7, 12pterq12, 15q25qter, 17q | 3, 13, 17p, X |
| 81 | F | 6.9 | Medulloblastoma | Posterior fossa | Yes | 1q, 5, 6, 8q | 7p, 21, X |
| 84 | F | 2.2 | Medulloblastoma | Posterior fossa | Yes | 7, 10p, 13, 14, 17 | 4, 8, 10q, 11, 15, 21, X |
| 113 | M | 1.4 | Medulloblastoma | Posterior fossa | Yes | 2, 6q10q24 | 16p, 17pterq23 |
| 164 | F | 11 | Medulloblastoma | Posterior fossa | Yes | | Normal |
| 207 | F | 1.2 | Medulloblastoma | Posterior fossa | Yes | | Normal |
| 212 | F | 3.9 | Medulloblastoma | Posterior fossa | Yes | 1031qter, 7q, 13q31qter, 17q | 10q, 17p, X |
| 214 | M | 0.7 | Medulloblastoma | Posterior fossa | Yes | | Normal |
| 227 | M | 9.5 | Medulloblastoma | Posterior fossa | Yes | 4q22qter, 17q | 9p13q21, 17p, 20 |

[a]Tumour sample was obtained at time of relapse. M, male; F, female.

specimens were fixed in acetic acid–formalin–ethanol (AFA; Carlo-Erba, Milan, Italy) and embedded in paraffin. Paraffin-embedded sections were then routinely stained with haematoxylin–eosin–saphranin (HES). Appropriate immunohistochemical analysis was performed when needed to rule out a differential diagnosis, especially for supratentorial tumours.

### Comparative genomic hybridization

CGH was performed as previously described (Paszek-Vigier et al, 1997). Briefly, tumour DNA was extracted using phenol–chloroform technique. Tumour DNA was labelled by nick translation with Texas red (TR)-dUTP (Dupont-NEN), whereas normal DNA from heathy male and female donors was labelled with fluorescein isothiocyanate (FITC)-dUTP (Dupont-NEN). A total of 150 ng of each donor's DNA and 20 µg of unlabelled Cot-1 DNA (Gibco, BRL) were ethanol precipitated, resuspended in Hybrisol VII (Oncor, Gaithersburg, MD, USA) and denatured for 10 min. After a 48-hour hybridization at 37°C, slides were washed for 5 min in 2 × saline–sodium citrate (SSC) at 73°C and examined using an epifluorescence microscope (DMRB, Leica). CGH analysis was performed using Powergene software (PSI, Houston, TX, USA). The over- and under-represented DNA segments were determined by calculating TR:FITC average ratio profiles. Average ratio images were calculated from at least six metaphases and had fixed thresholds. Chromosomal gains were considered when fluorescence ratios exceeded 1.15 and losses were considered for ratios lower than 0.85. Telomeric and heterochromatic regions were excluded from analysis, according to Kallioniemi et al (1994), as well as chromosomes 1p, 16q, 19, 22 and Y. Genomic amplification was defined by a red/green ratio > 1.5.

### RESULTS

CGH analysis was successfully performed for the 23 tested patients. Figure 1 summarizes the copy number changes detected in the 23 patients. Twenty contained aberrations in at least one

 *© Cancer Research Campaign 1999*

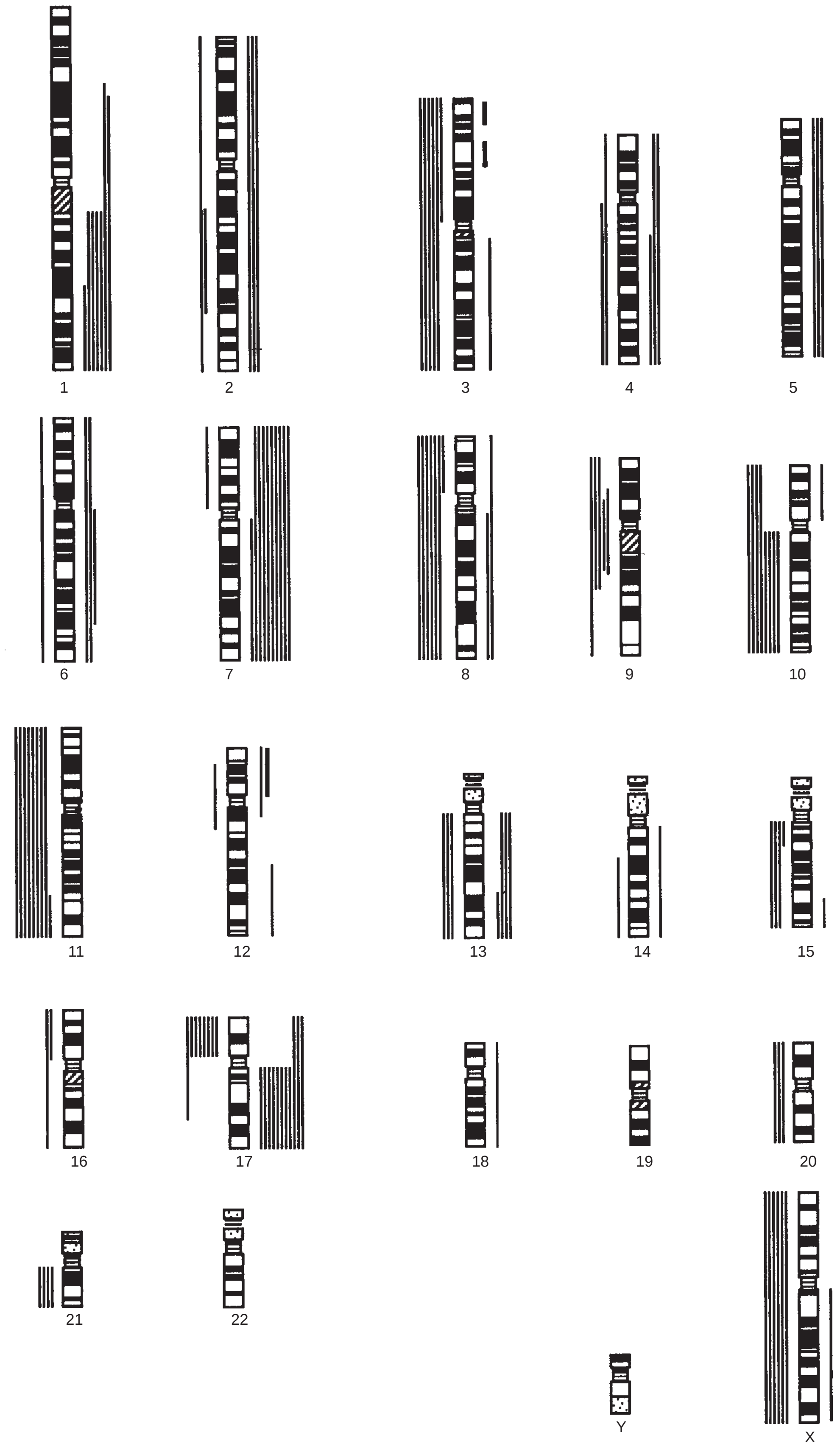

**Figure 1** Summary of the chromosomal gains and losses found in this series. Vertical lines on the right side of a chromosome represent a gain of genetic material and a vertical line on the left side a loss of material. Thick lines show genomic amplifications

© Cancer Research Campaign 1999

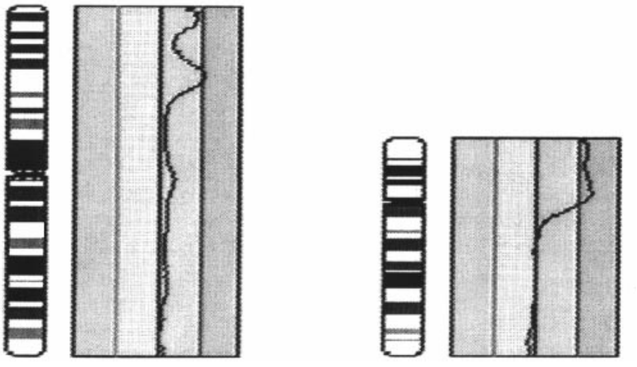

**Figure 2** A partial CGH 'karyotype' showing low-level amplifications on chromosomes 3 (averaged profile from eight copies of chromosome 3) and 12 (averaged profile from ten copies of chromosome 12) (patient 222). The median thick line represents the ratio 1, the left line the ratio 0.5 and the right line the ratio 1.5. This patient displays two different amplified regions on the short arm of chromosome 3 and multiple copy numbers of the short arm of chromosome 12

chromosomal region. The number of imbalances ranged from 0 to 13 (median = 6). The most frequent abnormality was a gain of 17q in 11 cases. Other recurrent abnormalities were a gain of chromosome 7 (nine patients), loss of 11 (eight patients), loss of 17p (eight patients), loss of 10q (eight patients), loss of chromosome X (six patients), loss of 8 (six patients), gain of 1q (six patients) and loss of chromosomes 3 and 9p (five patients each). The coexistence of gain of 17q and loss of 17p in six patients was suggestive of an isochromosome i(17q). Multiple copy numbers involving 3p and 12p were found in one patient (Figure 2). No amplification was found at other classical amplified loci (*MYC*, *MYCN* or *EGFR*). In the analyses, we also separated metastatic patients from children with localized disease. Among recurrent abnormalities described above, loss of chromosome 11 appeared to be less frequent in children with metastatic PNET.

### DISCUSSION

CGH has been used for the identification of chromosomal imbalances in a wide variety of solid tumours and haematologic malignancies. This technique circumvents many limitations associated with conventional cytogenetics, such as the difficulty to obtain metaphases in tumour cells or the often poor quality of chromosome spreads associated with the frequent high-complexity of chromosomal changes. Moreover, CGH does not require fresh tumour material, and even small pieces of tumour can be analysed. For all these reasons, CGH is a very powerful technique for the analysis of chromosomal changes in solid tumours. However, some limitations exist. Balanced chromosomal rearrangements are not detected and some regions, including 1p, 16q, 19, 22 and Y, are difficult to analyse. In this study, we detected chromosomal imbalances in 20 out of 23 tumours analysed. No abnormality was detected in three patients with metastatic disease, corresponding either to patients with a normal karyotype or only balanced structural abnormalities, or to patients with an important contamination with normal cells. Losses were slightly more frequent than gains (75 versus 60), and many recurrent chromosomal changes were found.

In agreement with published cytogenetic data, chromosome 17 was the most frequently involved. Gain of 17q was found in 11 patients (half of the abnormal patients) and loss of 17p was found in eight patients. In six cases, both abnormalities were present simultaneously, indicating the probable presence of an i(17q). Nevertheless, some patients have only gain of 17q and others have only loss of 17p, suggesting that these two abnormalities may involve different tumorigenic pathways. Recent data showed that different regions may be lost in 17p deletions, leading to the loss of different putative tumour suppressor genes (Biegel et al, 1992; Cogen et al, 1992; McDonald et al, 1994; Batra et al, 1995; Scheurlen et al, 1997). On the other hand, gain of chromosome 17q (through the formation of an isochromosome or an isolated gain of 17q) is probably an important second event, by modifying some gene dosage. This phenomenon has to be related to abnormalities described in another neuroectodermal tumour, i.e. neuroblastoma. Recently, it has been shown that gains of 17qter material were present in about 90% of high-grade neuroblastomas (Meddeb et al, 1996). Because neuroblastomas and medulloblastomas are both neuroectodermal tumours, a common tumorigenic event may be proposed, even if no candidate gene has so far been found.

We also found many other abnormalities, especially extra copies of chromosome 7 and losses of chromosomes 11 and 10q. Most of them are in agreement with data reported by Reardon et al (1997), but strongly differ from Schutz et al's data (1996). Both trisomy 7 and loss of chromosome 10 are the hallmark of another type of brain tumour, glioblastoma multiforme (Bigner et al, 1990). This tumour is especially frequent in adults, but rather rare in childhood. Moreover, embryologic origins of glioblastomas and medulloblastomas are totally different. The former is a glial tumour, whereas medulloblastoma is a neuroectodermal tumour. Are there common genetic abnormalities in childhood brain malignancies? This question has to be raised, but larger series will be needed to address this question.

Loss of 11q has already been reported in medulloblastomas (Callen et al, 1989; Vagner-Capodano et al, 1992; Reardon et al, 1997). Vagner-Capodano et al (1992) suggested that tumours with 11q abnormalities might constitute a subgroup of medulloblastoma different from tumours with an i(17q). Our results do not support this hypothesis. In our series, six patients had abnormalities of both chromosome 11 and 17; three with monosomy 11 and an i(17q) and three with monosomy 11 and gain of chromosome 17 or 17q. The loss of chromosome 11 was found to be more frequent in patients with localized disease (8/14 versus 1/9). Even if these respective frequencies are statistically different, a larger series would be necessary to conclude.

Other recurrent abnormalities were loss of chromosome X, 3, 8 and 9p–9q21–22, and gain of chromosome 1q. This latter abnormality is not a medulloblastoma-specific change since it has been described in a large variety of tumour types. Loss of chromosome 9p has been reported in adult gliomas, but not in childhood medulloblastoma. In adults, it seems to lead to p15/p16 deletions (Jen et al, 1994; Barker et al, 1997). Interestingly, the five patients with a chromosome 9 deletion share a loss of the q21–22 region. Recently, mutations of the PTCH gene, which maps at 9q22, have been reported in medulloblastoma (Pietsch et al, 1997; Wolter et al, 1997). We can speculate that these deletions in our patients include this gene. Losses in chromosome 3 have not been reported as non-random changes in medulloblastomas. We found loss of chromosome 3 in five patients, plus one patient with loss of 3p and

*© Cancer Research Campaign 1999*

gain of 3q (suggesting the presence of an isochromosome 3q). The entire chromosome 8 was lost in six patients and another patient exhibited loss of chromosome 8p. These losses of chromosome 8 material are in agreement with Reardon et al's data (1997), which found a recurrent loss of the short arm of chromosome 8. Many genes localized on 3p or 8p could potentially be the main target of these deletions, and more patients will be necessary to try to define the critical minimal deleted region.

No high-level amplification has been found in this study. Only one patient displayed multiple copy number changes: two gains on 3p (3p21 and 3p25) and one gain of the entire chromosome 12 short arm. In these three cases, deviations from the baseline are compatible with multiple copy numbers. These regions have not been previously reported. Amplifications have rarely been reported in medulloblastomas, usually as double minutes. When analysed, the target genes of these amplifications were either *MYC*, *MYCN* or *EGFR* (Rouah et al, 1989; Wasson et al, 1990; Fuller and Bigner, 1992), and more recently the 5p15 and 11q22 regions (Reardon et al, 1997). None of these regions was amplified in our series.

In conclusion, this CGH study identified many non-random copy number changes, confirming data from the Reardon group (1997). Larger series of consecutive patients would define the exact frequency and significance of these individual chromosomal abnormalities. This analysis prompts the elaboration of large prospective studies in order to determine the potential prognostic significance of these copy number changes.

### ACKNOWLEDGEMENTS

We thank Professor Alain Pierre-Kahn, Professor Christian Sainte-Rose and all the staff of the Department of Pediatric Neurosurgery operating theater (Hopital Necker) for their help in providing us with brain tumour tissue samples. This study was supported by the Comite de Loire-Atlantique de la Ligue contre le Cancer.

### REFERENCES

Badiali M, Pession A, Basso G, Andreini L, Rigobello L, Galassi E and Giangaspero F (1995) N-myc and c-myc oncogenes amplification in medulloblastoma: evidence of particularly aggressive behaviour of a tumor with c-myc amplification. *Tumori* **77**: 118–121

Barker FG, Chen P, Furman F, Aldape KD, Edwards MS and Israel MA (1997) P16 deletion and mutation analysis in human brain tumors. *J Neurdoncol* **31**: 17–23

Batra S, McLendon R, Koo JS, Castelino-Prabhu S, Fuchs H, Krischer J, Friedman H, Bigner D and Bigner H (1995) Prognostic implications of chromosome 17p deletions in human medulloblastomas. *J Neurooncol* **24**: 39–45

Biegel JA, Rorke LB, Packer RJ, Sutton LN, Schut L, Bonner K and Emanuel BS (1989) Isochromosome 17q in primitive neuroectodermal tumors of the central nervous system. *Genes Chromosomes Cancer* **1**: 139–147

Biegel JA, Burk CD, Barr FG and Emanuel BS (1992) Evidence for a 17p tumor-related locus distinct from p53 in pediatric primitive neuroectodermal tumors. *Cancer Res* **52**: 2347–2350

Biegel JA, Rorke LB, Janss AJ, Sutton LN and Parmiter AH (1995) Isochromosome 17q demonstrated by interphase fluorescence in situ hybridization in primitive neuroectodermal tumors of the central nervous system. *Genes Chromosomes Cancer* **14**: 85–96

Bigner SH, Mark J, Friedman HS, Biegel JA and Bigner DD (1988) Structural chromosomal abnormalities in human medulloblastoma. *Cancer Genet Cytogenet* **30**: 91–101

Bigner SH, Mark J and Bigner DD (1990) Cytogenetics of human brain tumors. *Cancer Genet Cytogenet* **47**: 141–154

Callen DF, Circocco L and Moore L (1989) A der(11)t(8;11) in two medulloblastomas: a possible non-random cytogenetic abnormality. *Cancer Genet Cytogenet* **38**: 255–260

Cogen PH, Daneshvar L, Metzger AK, Duyk G, Edwards MS and Sheffield VC (1992) Involvement of multiple 17p loci in medulloblastoma tumorigenesis. *Am J Hum Genet* **50**: 584–589

Farwell JR, Dohrmann GJ and Flanner JT (1977) Central nervous system tumors in children. *Cancer* **40**: 3123–3132

Fujii Y, Hongo T and Hayashi Y (1994) Chromosome analysis of brain tumors in childhood. *Genes Chromosomes Cancer* **11**: 205–215

Fuller GN and Bigner SH (1992) Amplified cellular oncogenes in neoplasms of the human nervous system. *Mutat Res* **276**: 299–306

Griffin CA, Hawkins AL, Packer FJ, Rorke LB and Emanuel BS (1988) Chromosome abnormalities in pediatric brain tumors. *Cancer Res* **48**: 175–180

Jen J, Harper JW, Bigner SH, Bigner DD, Papadopoulos N, Markowitz S, Willson JK, Kinzler KW and Vogelstein B (1994) Deletion of p16 and p15 genes in brain tumors. *Cancer Res* **54**: 6353–6358

Kallioniemi OP, Kallioniemi A, Piper J, Isola J, Waldman FM, Gray JW and Pinkel D (1994) Optimizing comparative genomic hybridization for analysis of DNA sequence copy number changes in solid tumors. *Genes Chromosomes Cancer* **10**: 231–243

Karnes PS, Tuan NT, Cui MY, Raffel C, Gilles FH, Barranger JA and Ying KL (1992) Cytogenetic analysis of 39 pediatric central nervous system tumors. *Cancer Genet Cytogenet* **59**: 12–19

McDonald J, Daneshvar L, Willert J, Matsumara K, Waldman F and Cogen P (1994) Physical mapping of chromosome 17p 13.3 in the region of a putative tumor suppressor gene important in medulloblastoma. *Genomics* **23**: 229–232

Meddeb M, Danglot G, Chudoba I, Venuat AM, Bénard J, Avet-Loiseau H, Vasseur B, Le Paslier D, Terrier-Lacombe MJ, Hartmann O and Bernheim A (1996) Additional copies of a 25 Mb chromosomal region originating from 17q23. 1-qter are present in 90% of high-grade neuroblastomas. *Genes Chromosomes Cancer* **17**: 156–165

Neumann E, Kalousek D, Norman M, Steinbok P, Cochrane D and Goddard K (1993) Cytogenetic analysis of 109 pediatric central nervous system tumors. *Cancer Genet Cytogenet* **71**: 40–49

Paszek-Vigier M, Talmant P, Méchinaud F, Garand R, Harousseau JL, Bataille R and Avet-Loiseau H (1997) Comparative genomic hybridization is a powerful tool, complementary to cytogenetics, to identify chromosomal abnormalities in childhood acute lymphoblastic leukemia. *Br J Haematol* **99**: 589–596

Pietsch T, Waha A, Koch A, Kraus J, Albrecht S, Tonn J, Sorensen N, Berthold F, Henk B, Schmandt N, Wolf HK, von Deimling A, Wainwright B, Chenevix-Trench G, Wiestler OD and Wicking C (1997) Medulloblastomas of the desmoplastic variant carry mutations of the human homologue of Drosophila patched. *Cancer Res* **57**: 2085–2088

Reardon DA, Michalkiewicz E, Boyett JM, Sublett JE, Entrekin RE, Ragsdale ST, Valentine MB, Behm FG, Li H, Heideman RL, Kun LE, Shapiro DN and Look AT (1997) Extensive genomic abnormalities in childhood medulloblastoma by comparative genomic hybridization. *Cancer Res* **57**: 4042–4047

Rouah E, Wilson DR, Armstrong DL and Darlington GJ (1989) N-myc amplification and neuronal differentiation in human primitive neuroectodermal tumors of the central nervous system. *Cancer Res* **49**: 1797–1801

Scheurlen WG, Seranski P, Mincheva A, Kuhl J, Sorensen N, Krauss J, Lichter P, Poustka A and Wilgenbus KK (1997) High-resolution mapping of chromosome arm 17p in childhood primitive neuroectodermal tumors reveals a common chromosomal disruption within the the Smith-Magenis region, an unstable region in chromosome band 17p11.2. *Genes Chromosomes Cancer* **18**: 50–58

Schutz BR, Scheurlen W, Krauss J, Du Manoir S, Joos S, Bentz M and Lichter P (1996) Mapping of chromosomal gains and losses in primitive neuroectodermal tumors by comparative genomic hybridization. *Genes Chromosomes Cancer* **16**: 196–203

Vagner-Capodano AM, Gentet JC, Gambarelli D, Pellissier JF, Gouzien M, Lena G, Genitori L, Choux M and Raybaud C (1992) Cytogenetic studies in 45 pediatric brain tumors. *Pediatr Hematol Oncol* **9**: 223–235

Wasson JC, Saylors III LS, Zeltzer P, Friedman HS, Bigner SH, Burger PC, Bigner DD, Look AT, Douglass EC and Brodeur GM (1990) Oncogene amplification in pediatric brain tumors. *Cancer Res* **50**: 2987–2990

Wolter M, Reifenberger J, Sommer C, Ruzicka T and Reifenberger G (1997) Mutations in the human homologue of the Drosophila segment polarity gene patched (PTCH) in sporadic basal cell carcinomas of the skin and primitive neuroectodermal tumors of the central nervous system. *Cancer Res* **57**: 2581–2585

© Cancer Research Campaign 1999

## References

[PDF_00294] Badiali M., Pession A., Basso G., Andreini L., Rigobello L., Galassi E., Giangaspero F. (1991). N-myc and c-myc oncogenes amplification in medulloblastomas. Evidence of particularly aggressive behavior of a tumor with c-myc amplification.. Tumori.

[PDF_00298] Barker F. G., Chen P., Furman F., Aldape K. D., Edwards M. S., Israel M. A. (1997). P16 deletion and mutation analysis in human brain tumors.. J Neurooncol.

[PDF_00300] Batra S. K., McLendon R. E., Koo J. S., Castelino-Prabhu S., Fuchs H. E., Krischer J. P., Friedman H. S., Bigner D. D., Bigner S. H. (1995). Prognostic implications of chromosome 17p deletions in human medulloblastomas.. J Neurooncol.

[PDF_00309] Biegel J. A., Rorke L. B., Janss A. J., Sutton L. N., Parmiter A. H. (1995). Isochromosome 17q demonstrated by interphase fluorescence in situ hybridization in primitive neuroectodermal tumors of the central nervous system.. Genes Chromosomes Cancer.

[PDF_00303] Biegel J. A., Rorke L. B., Packer R. J., Sutton L. N., Schut L., Bonner K., Emanuel B. S. (1989). Isochromosome 17q in primitive neuroectodermal tumors of the central nervous system.. Genes Chromosomes Cancer.

[PDF_00316] Bigner S. H., Mark J., Bigner D. D. (1990). Cytogenetics of human brain tumors.. Cancer Genet Cytogenet.

[PDF_00313] Bigner S. H., Mark J., Friedman H. S., Biegel J. A., Bigner D. D. (1988). Structural chromosomal abnormalities in human medulloblastoma.. Cancer Genet Cytogenet.

[PDF_00318] Callen D. F., Cirocco L., Moore L. (1989). A der(11)t(8;11) in two medulloblastomas. A possible nonrandom cytogenetic abnormality.. Cancer Genet Cytogenet.

[PDF_00321] Cogen P. H., Daneshvar L., Metzger A. K., Duyk G., Edwards M. S., Sheffield V. C. (1992). Involvement of multiple chromosome 17p loci in medulloblastoma tumorigenesis.. Am J Hum Genet.

[PDF_00324] Farwell J. R., Dohrmann G. J., Flannery J. T. (1977). Central nervous system tumors in children.. Cancer.

[PDF_00326] Fujii Y., Hongo T., Hayashi Y. (1994). Chromosome analysis of brain tumors in childhood.. Genes Chromosomes Cancer.

[PDF_00328] Fuller G. N., Bigner S. H. (1992). Amplified cellular oncogenes in neoplasms of the human central nervous system.. Mutat Res.

[PDF_00330] Griffin C. A., Hawkins A. L., Packer R. J., Rorke L. B., Emanuel B. S. (1988). Chromosome abnormalities in pediatric brain tumors.. Cancer Res.

[PDF_00332] Jen J., Harper J. W., Bigner S. H., Bigner D. D., Papadopoulos N., Markowitz S., Willson J. K., Kinzler K. W., Vogelstein B. (1994). Deletion of p16 and p15 genes in brain tumors.. Cancer Res.

[PDF_00335] Kallioniemi O. P., Kallioniemi A., Piper J., Isola J., Waldman F. M., Gray J. W., Pinkel D. (1994). Optimizing comparative genomic hybridization for analysis of DNA sequence copy number changes in solid tumors.. Genes Chromosomes Cancer.

[PDF_00339] Karnes P. S., Tran T. N., Cui M. Y., Raffel C., Gilles F. H., Barranger J. A., Ying K. L. (1992). Cytogenetic analysis of 39 pediatric central nervous system tumors.. Cancer Genet Cytogenet.

[PDF_00342] McDonald J. D., Daneshvar L., Willert J. R., Matsumura K., Waldman F., Cogen P. H. (1994). Physical mapping of chromosome 17p13.3 in the region of a putative tumor suppressor gene important in medulloblastoma.. Genomics.

[PDF_00345] Meddeb M., Danglot G., Chudoba I., Vénuat A. M., Bénard J., Avet-Loiseau H., Vasseur B., Le Paslier D., Terrier-Lacombe M. J., Hartmann O. (1996). Additional copies of a 25 Mb chromosomal region originating from 17q23.1-17qter are present in 90% of high-grade neuroblastomas.. Genes Chromosomes Cancer.

[PDF_00350] Neumann E., Kalousek D. K., Norman M. G., Steinbok P., Cochrane D. D., Goddard K. (1993). Cytogenetic analysis of 109 pediatric central nervous system tumors.. Cancer Genet Cytogenet.

[PDF_00353] Paszek-Vigier M., Talmant P., Méchinaud F., Garand R., Harousseau J. L., Bataille R., Avet-Loiseau H. (1997). Comparative genomic hybridization is a powerful tool, complementary to cytogenetics, to identify chromosomal abnormalities in childhood acute lymphoblastic leukaemia.. Br J Haematol.

[PDF_00359] Pietsch T., Waha A., Koch A., Kraus J., Albrecht S., Tonn J., Sörensen N., Berthold F., Henk B., Schmandt N. (1997). Medulloblastomas of the desmoplastic variant carry mutations of the human homologue of Drosophila patched.. Cancer Res.

[PDF_00362] Reardon D. A., Michalkiewicz E., Boyett J. M., Sublett J. E., Entrekin R. E., Ragsdale S. T., Valentine M. B., Behm F. G., Li H., Heideman R. L. (1997). Extensive genomic abnormalities in childhood medulloblastoma by comparative genomic hybridization.. Cancer Res.

[PDF_00366] Rouah E., Wilson D. R., Armstrong D. L., Darlington G. J. (1989). N-myc amplification and neuronal differentiation in human primitive neuroectodermal tumors of the central nervous system.. Cancer Res.

[PDF_00369] Scheurlen W. G., Seranski P., Mincheva A., Kühl J., Sörensen N., Krauss J., Lichter P., Poustka A., Wilgenbus K. K. (1997). High-resolution deletion mapping of chromosome arm 17p in childhood primitive neuroectodermal tumors reveals a common chromosomal disruption within the Smith-Magenis region, an unstable region in chromosome band 17p11.2.. Genes Chromosomes Cancer.

[PDF_00374] Schütz B. R., Scheurlen W., Krauss J., du Manoir S., Joos S., Bentz M., Lichter P. (1996). Mapping of chromosomal gains and losses in primitive neuroectodermal tumors by comparative genomic hybridization.. Genes Chromosomes Cancer.

[PDF_00378] Vagner-Capodano A. M., Gentet J. C., Gambarelli D., Pellissier J. F., Gouzien M., Lena G., Genitori L., Choux M., Raybaud C. (1992). Cytogenetic studies in 45 pediatric brain tumors.. Pediatr Hematol Oncol.

[PDF_00381] Wasson J. C., Saylors R. L., Zeltzer P., Friedman H. S., Bigner S. H., Burger P. C., Bigner D. D., Look A. T., Douglass E. C., Brodeur G. M. (1990). Oncogene amplification in pediatric brain tumors.. Cancer Res.

[PDF_00384] Wolter M., Reifenberger J., Sommer C., Ruzicka T., Reifenberger G. (1997). Mutations in the human homologue of the Drosophila segment polarity gene patched (PTCH) in sporadic basal cell carcinomas of the skin and primitive neuroectodermal tumors of the central nervous system.. Cancer Res.

